# Extraction of phenolic compounds from *Moringa oleifera* Lam. leaves with ultrasonic-assisted deep eutectic solvents

**DOI:** 10.3389/fnut.2024.1405128

**Published:** 2024-08-12

**Authors:** Zilin Wang, Yang Tian, Min Yang, Junyan Yang, Yifan Wang, Liang Tao, Jun Sheng, Chongying Shi

**Affiliations:** ^1^College of Food Science and Technology, Yunnan Agricultural University, Kunming, China; ^2^National Research and Development Professional Center for Moringa Processing Technology, Yunnan Agricultural University, Kunming, China; ^3^Engineering Research Center of Development and Utilization of Food and Drug Homologous Resources, Ministry of Education, Yunnan Agricultural University, Kunming, China; ^4^Yunnan Key Laboratory of Precision Nutrition and Personalized Food Manufacturing, Yunnan Agricultural University, Kunming, China; ^5^Puer University, Puer, China

**Keywords:** deep eutectic solvents, *Moringa oleifera* Lam. leaves, optimization of extraction process, phenolic compounds, stability and antioxidant activity, ultrasound assisted extraction

## Abstract

**Background:**

In this study, deep eutectic solvents (DESs) combined with ultrasound-assisted extraction (UAE) were used to extract bioactive compounds from the leaves of *Moringa oleifera* Lam.

**Methods:**

The FT-IR method was used to analyze the structural characteristics of the DESs, and the extraction efficiencies of the DESs for total phenolic content (TPC) and total flavonoid content (TFC) were evaluated. The stability of the extracts under high temperature and UV radiation was assessed, and their antioxidant activity was investigated after undergoing *in vitro* simulated digestion.

**Results:**

The results show that the seven DESs extracted more TPC and TFC than did the 70% ethanol (36.27 ± 1.58 mg GAE/g, 23.09 ± 1.47 mg RT/g), and the extraction process of UAE-DES was optimized by selecting choline chloride: citric acid as the DES solvent, which has the highest extraction of TPC (86.92 ± 1.34 mg GAE/g) and TFC (49.73 ± 0.85 mg RT/g). The stability results indicated that the DES phenolic extracts were less stable when exposed to high temperature and UV radiation, indicating that DES extracts have better bioactivity. Moreover, after *in vitro* simulated digestion, the DES extract shows a higher DPPH free radical scavenging capacity (12.79 ± 3.88 mmol Trolox/g of DES extracts, 6.99 ± 4.02 mmol Trolox/g of ethanol extracts) and ferric ion reducing antioxidant power (62.61 ± 1.71 mmol Trolox/g of DES extracts, 55.07 ± 1.66 mmol Trolox/g of ethanol extracts) than ethanol extracts.

**Conclusion:**

This study confirmed that DESs are a new and environmentally friendly solvent that can be used for the extraction of phenolic compounds.

## Graphical abstract

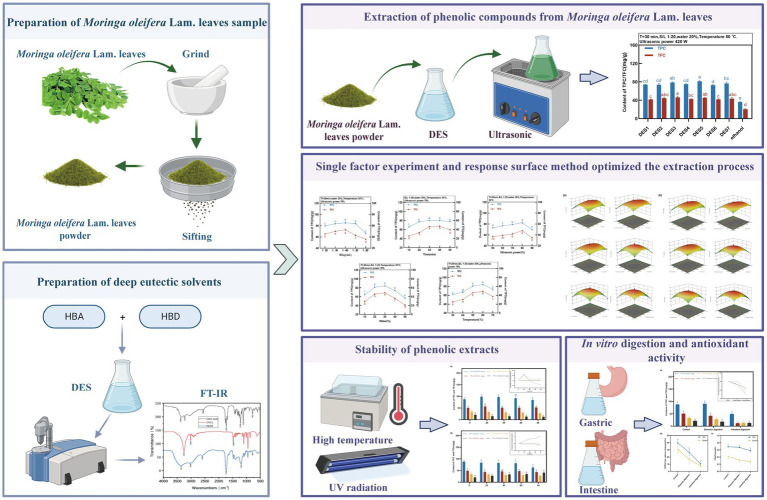

Created with Biorender.com.

## 1 Introduction

*Moringa oleifera* Lam. (MOL), which belongs to the Moringaceae family, is widely cultivated in tropical and subtropical regions of countries such as India and Africa for use as a nutritional supplement in medicine and food applications and is recognized as a “magic tree” (1). Phenolic compounds play a crucial role in maintaining human health, and there is increasing interest in using diet to enhance health (2). Studies have suggested that MOL is a valuable plant species, especially *Moringa oleifera* Lam. leaves (MOLLs) contain significant amounts of phenolic compounds, making them ideal for extraction (3). The Ministry of Health of the People’s Republic of China approved the use of MOLLs as a new source of food in 2012.^1^ The potential applications of MOLLs in the food industry have been expanded by this decision.

Phenolic compounds are secondary metabolites that are commonly found in plants such as vegetables, fruits, and grains (4, 5). They are recognized for their antioxidant properties and play a significant role as antioxidants (4). In previous studies, various methods have been employed to extract phenolic compounds, such as organic reagent extraction, physical-assisted extraction through the use of ultrasound, microwaves, high pressure, etc., and enzyme-assisted extraction (6). Moreover, it is crucial to select an appropriate solvent that determines the success of extracting phenolic compounds. Hydrogen bonding within a solvent is a critical factor that influences the rate of phenolic compound diffusion (7). Thus, it is vital to carefully choose suitable solvents and extraction techniques to improve the effectiveness of phenolic compounds in MOLLs.

Deep eutectic solvents (DESs) are a new type of environmentally friendly solvent that involves strong hydrogen bonding between hydrogen bond donors (HBDs) and hydrogen bond acceptors (HBAs) (8). Studies have shown that compared with organic reagents, DESs significantly improve the extraction efficiency of bioactive compounds because DESs can efficiently dissolve cell walls and enable effective intermolecular interactions between the solvent and the cellulose chain in plants (9). The application of DESs as eco-friendly extraction solvents has become popular due to their easy manufacturing and favorable environmental impact. Ultrasound-assisted extraction (UAE) utilizes ultrasound-induced cavitation to enhance the breakdown of plant cell walls (10). Thus, UAE enables the rapid extraction of additional phenolic compounds in a shorter time frame. While recent studies have analyzed the impact of DES solvents on phenolic compound extraction proficiency, further investigation into the stability and activity of phenolic compounds after DES extraction is necessary to provide additional evidence of the effectiveness of DESs in phenolic compound extraction.

The purpose of this study was to evaluate the effectiveness of DESs in extracting phenolic compounds from MOLLs. This research examined the performance of different DESs using choline chloride (ChChl) as an HBA and alcohols, organic acids, amides, and sugars as HBDs in the extraction of phenolic compounds from MOLLs. The most efficient DES was employed for extracting phenolic compounds through one-factor and response surface methodology (RSM) for UAE-DES optimized extraction. Furthermore, the high temperature and UV radiation stability of the DES extract and the antioxidant activity after simulated *in vitro* digestion were measured to demonstrate that DES is a suitable solvent for the extraction of bioactive compounds.

## 2 Materials and methods

### 2.1 Raw material

The MOLLs used in the experiment were obtained from Yunnan Tianyou Technology Development Co., Ltd. The leaves were dried in an oven at 55°C until they reached a consistent weight, pulverized into a fine powder using an 80-mesh stainless steel sieve and stored at 4°C.

### 2.2 Chemicals and reagents

The chemicals used in the study were obtained from the suppliers described below: choline chloride (ChChl) (≥98%), gallic acid (≥98%), rutin (≥98%), and Folin–Ciocalteu reagent were purchased from Beijing Solarbio Science & Technology Co., Ltd. (Beijing, China). Urea (≥98%), levulinic acid (≥98%), glycerol (≥99%), sodium phosphate monobasic dihydrate (NaH_2_PO_4_·2H_2_O) (≥99%), sodium phosphate dibasic dihydrate (Na_2_HPO_4_·2H_2_O) (≥98%), bile salt (pig), potassium phosphate monobasic (KH_2_PO_4_) (≥99%), magnesium chloride hexahydrate (MgCl2·6H2O) (≥98%), and ammonium carbonate (≥99%) were purchased from Shanghai Yuanye Bio-Technology Co., Ltd. (Shanghai, China). Ethylene glycol (≥99%), lactic acid (≥85%), citric acid (≥99.5%), 2,2′-diphenyl-1-picrylhydrazyl (DPPH), potassium ferricyanide (K3FeC6N6) (≥99%), trichloroacetic acid (≥99%), iron chloride (≥99.9%), pepstatin, trypsin, calcium chloride (CaCl_2_) (≥99.9%), potassium chloride (KCl) (≥99.5%), α-D-glucose (≥96%), sodium carbonate (Na_2_CO_3_) (≥99.5%), sodium nitrite (NaNO_2_) (≥99%), aluminum nitrate nonahydrate (Al(NO_3_)_3_) (≥99%), sodium hydroxide (NaOH) (≥97%), sodium bicarbonate (NaHCO_3_) (≥99%), and sodium chloride (NaCl) (≥99%) were purchased from Shanghai Aladdin Bio-Chem Technology Co., Ltd. (Shanghai, China). Ethyl alcohol (≥99%), hydrochloric acid (HCl), and phosphoric acid (H_3_PO_4_) were purchased from Chengdu Chron Chemical Reagent Co., Ltd. (Chengdu, China).

### 2.3 Preparation of DESs

Seven DESs were prepared according to previous methods (11) and labeled DES1–DES7. Briefly, the mass of two specific substances was calculated, following the molar ratios of different HBA and HBD. Then, the two components were mixed in a beaker containing 20% (w/w) water to reduce the viscosity of the DES. The beaker was stored in a water bath at 80°C for 2 h until the solution became transparent. Table 1 displays the DES combinations used in this study.

**Table 1:** DESs and their molar ratio, acronym, color, and density (measured at room temperature and a water content of 20%).

Acronym	HBA	Molecular structure	HBD	Molecular structure	Molar ratio	Color of solution	Density (g/cm^3^)
DES-1	Choline chloride	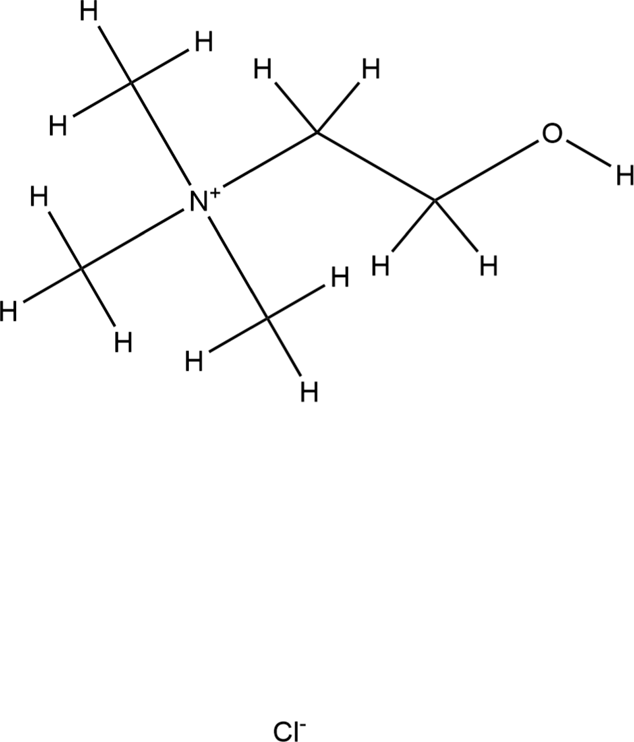	Urea	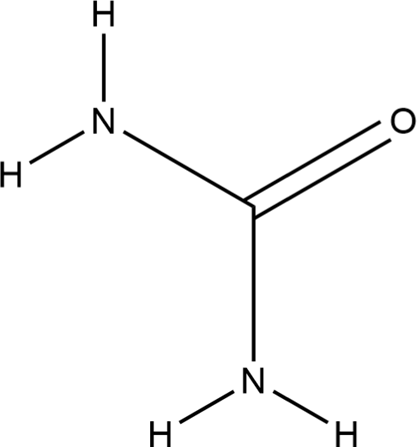	1:2	Colorless	1.015 ± 0.06
DES-2			Ethylene glycol	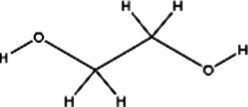	1:2	Colorless	0.903 ± 0.02
DES-3			Levulinic acid	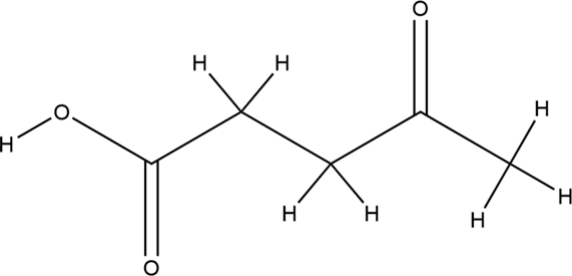	1:1	Light yellow color	0.953 ± 0.07
DES-4			Glucose	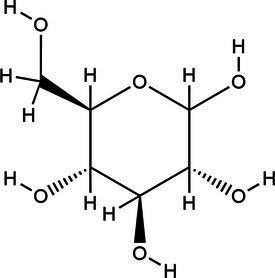	3:2	Light yellow color	0.973 ± 0.08
DES-5			Citric acid	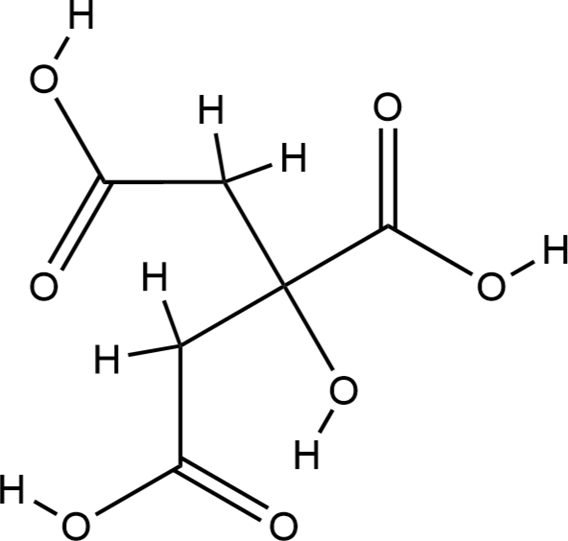	1:1	Light yellow color	0.966 ± 0.06
DES-6			Glycerin	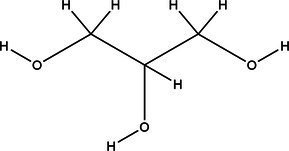	1:2	Colorless	0.907 ± 0.08
DES-7			Lactic acid	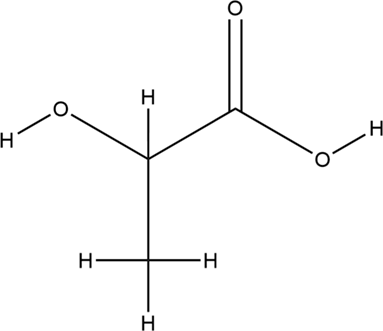	1:3	Colorless	0.945 ± 0.05

The chemical structure of the 2D structure was determined by the PubChem (https://pubchem.ncbi.nlm.nih.gov/) query through ChemDraw export.

### 2.4 Characterization of the DESs

The density (g/cm^3^) of the DES was determined by measuring the volume of the DES solution (*V*) at room temperature. The mass of the DES at that volume (*M*) was then determined using an experimental balance.

The prepared DES1-DES7 was analyzed using an FT-IR system (Thermo Nicolet IS 5, United States) that obtained 32 infrared scans at a resolution of 4 cm^−1^ (12).

### 2.5 Extraction of phenolic compounds from MOLLs

The MOLLs were dried, ground, and sifted before they were set aside. Powdered MOLLs and various DESs were mixed in a beaker at a solid–liquid ratio of 1:20 g/mL and agitated thoroughly. A control group was established by using the 70% ethanol extraction method. The ultrasonic power was set to 420 W, and the temperature was maintained at 50°C. The extract was centrifuged (5,000 rpm, 10 min) after 30 min, and the amount of supernatant was measured.

### 2.6 Assays of total phenol content and total flavonoid content

Total phenol content (TPC) was determined as previously documented and with appropriate modifications (13). The extract was diluted six-fold. A 0.5 mL aliquot of the diluted sample was mixed with 1.5 mL of 0.25 mol/L Folin–Ciocalteu reagent. Afterward, 3 mL of 75 g/L Na_2_CO_3_ was added and mixed, and the mixture was made up to 10 mL with ultrapure water. The reaction mixture was left to stand for 60 min away from light, after which the absorbance at 760 nm was measured. The total phenolic content of the sample was determined using gallic acid as a standard. Using gallic acid as a standard, the calibration curve equations is: *y* = 0.860*x* − 0.0449, *R*^2^ = 0.9989.

The total flavonoid content of the MOLLs was determined using the aluminum trichloride method (14). The sample extract was diluted six times before transferring 2 mL of the extract into a 25 mL triangular conical flask. After adding 1 mL of a 5% NaNO_2_ solution, the solution was mixed thoroughly and left to stand for 6 min. Next, 1 mL of 10% Al(NO_3_)_3_ solution was added to the mixture and mixed thoroughly, followed by the addition of 10 mL of a 4% NaOH solution. The volume of the solution was adjusted to 25 mL with ultrapure water before the solution was allowed to stand for 15 min. Rutin was used as the standard for calculating the total flavonoid content in the samples. Using rutin as a standard, the calibration curve equations is: *y* = 0.501*x* + 0.089, *R*^2^ = 0.9938.

### 2.7 Process optimization of ultrasound-assisted DES extraction of phenolic compounds from MOLLs

#### 2.7.1 Single-factor experiment

The effects of the solid–liquid ratio (1:20, 1:30, 1:40, 1:50, and 1:60 g/mL), extraction time (10, 20, 30, 40, and 50 min), water content (10, 20, 30, 40, and 50%), temperature (30, 40, 50, 60, and 70°C), and ultrasonic power (300, 360, 420, 480, and 540 W) on the content of phenolic compounds extracted by the DES from the MOLLs were investigated by taking the TPC and total flavonoid content (TFC) extracted as the evaluation indices.

#### 2.7.2 Response surface optimization experiment

Box–Behnken design (BBD) in RSM can determine the optimal process conditions using fewer experiments. It has been widely used to explore the optimization process of extraction (15). The extraction time, water content, ultrasonic power, and temperature were designated as the influential factors responsible for the optimization of the response surface based on the results of the single-factor experiment. The TPC and TFC were chosen as the response values, and a 4-factor and 3-level experiment was conducted to optimize the process of DES extraction of phenolic compounds from MOLLs using BBD design. The factor levels are shown in Table 2.

**Table 2:** Actual values from the BBD design.

Runs	Factors				Actual values	
	*A* Extraction time (min)	*B* Water content (%)	*C* Ultrasonic power (W)	*D* Temperature (°C)	TPC (mg GAE/g)	TFC (mg RT/g)
1	20 (−1)	20 (−1)	80 (0)	60 (0)	88.1 ± 1.43	51.30 ± 0.85
2	30 (0)	30 (0)	80 (0)	60 (0)	79.07 ± 3.57	43.83 ± 1.40
3	40 (1)	30 (0)	80 (0)	50 (−1)	72.89 ± 1.11	36.35 ± 1.46
4	30 (0)	40 (1)	70 (−1)	60 (0)	73.51 ± 0.90	37.56 ± 3.10
5	30 (0)	30 (0)	70 (−1)	70 (1)	67.75 ± 3.85	33.59 ± 2.56
6	30 (0)	20 (−1)	80 (0)	50 (−1)	72.15 ± 1.10	36.31 ± 1.22
7	30 (0)	30 (0)	80 (0)	60 (0)	75.5 ± 3.12	40.17 ± 1.12
8	30 (0)	20 (−1)	70 (−1)	60 (0)	78.52 ± 1.48	42.33 ± 2.50
9	20 (−1)	40 (1)	80 (0)	60 (0)	74.41 ± 0.86	38.72 ± 0.81
10	30 (0)	40 (1)	80 (0)	70 (1)	87.09 ± 0.94	50.79 ± 2.17
11	30 (0)	40 (1)	90 (1)	60 (0)	87.26 ± 2.36	51.45 ± 0.64
12	30 (0)	30 (0)	90 (1)	70 (1)	70.28 ± 0.54	34.82 ± 1.22
13	20 (−1)	30 (0)	80 (0)	70 (1)	72.89 ± 1.94	37.50 ± 1.09
14	40 (1)	20 (−1)	80 (0)	60 (0)	76.03 ± 0.64	40.14 ± 0.92
15	40 (1)	40 (1)	80 (0)	60 (0)	78.74 ± 1.05	43.40 ± 2.09
16	30 (0)	30 (0)	80 (0)	60 (0)	87.75 ± 0.55	50.94 ± 1.48
17	30 (0)	30 (0)	80 (0)	60 (0)	77.44 ± 1.45	40.85 ± 2.64
18	30 (0)	30 (0)	90 (1)	50 (−1)	80.57 ± 0.68	44.76 ± 1.24
19	30 (0)	40 (1)	80 (0)	50 (−1)	79.75 ± 0.98	43.73 ± 2.31
20	20 (−1)	30 (0)	90 (1)	60 (0)	81.72 ± 1.54	46.27 ± 0.97
21	40 (1)	30 (0)	80 (0)	70 (1)	74.92 ± 1.43	38.94 ± 3.72
22	30 (0)	30 (0)	80 (0)	60 (0)	81.66 ± 2.09	45.66 ± 1.08
23	40 (1)	30 (0)	90 (1)	60 (0)	74.99 ± 1.50	39.32 ± 1.12
24	30 (0)	30 (0)	70 (−1)	50 (−1)	88.1 ± 1.43	51.30 ± 0.85
25	20 (−1)	30 (0)	70 (−1)	60 (0)	79.07 ± 3.57	43.83 ± 1.40
26	40 (1)	30 (0)	70 (−1)	60 (0)	72.89 ± 1.11	36.35 ± 1.46
27	30 (0)	20 (−1)	80 (0)	70 (1)	73.51 ± 0.90	37.56 ± 3.10
28	20 (−1)	30 (0)	80 (0)	50 (−1)	67.75 ± 3.85	33.59 ± 2.56
29	30 (0)	20 (−1)	90 (1)	60 (0)	72.15 ± 1.10	36.31 ± 1.22

### 2.8 Evaluation of the stability and antioxidant activity of phenolic extracts

#### 2.8.1 Stability of phenolic extracts under high temperature and UV radiation

High temperature: One gram each of DES and ethanol extract samples was dissolved in 1 mL of water and placed in 5 mL test tubes, which were kept at 100°C in a water bath for 0 min, 20 min, 40 min, 60 min, and 80 min, and samples were taken to determine the TPC and TFC content and to observe the changes in the phenolic content of the MOLLs at high temperature by the two extraction methods.

UV radiation: One gram of each sample of DES and ethanol extract was dissolved in 1 mL of water, placed in a glass dish and kept under UVA and UVB for 0 min, 20 min, 40 min, 60 min, or 80 min. Samples were taken to determine the TPC and TFC contents and to observe the effects of UV radiation on the phenolic content of the MOLLs by the two extraction methods.

#### 2.8.2 *In vitro* gastrointestinal simulation of digestion and detection of antioxidant activity

##### 2.8.2.1 *In vitro* gastrointestinal simulation of digestion

The stability of the MOLL phenolic compound digestion process was evaluated by simulating *in vitro* gastrointestinal digestion using the INFOGEST 2.0 method (16). The digestion process involved two phases: the stomach and small intestine. Table 3 provides the procedure for preparing both the stomach digestion reserve solution and the small intestine digestion reserve solution.

**Table 3:** Preparation of the *in vitro* digestion.

Reagent	Concentration	Gastric digestive storage solution/mL	Small bowel digestive storage solution/mL
KCl	37.3 g/L	6.9	6.8
KH_2_PO_4_	68 g/L	0.9	0.8
NaHCO_3_	84 g/L	12.5	42.5
NaCl	117 g/L	11.8	9.6
Mg_2_Cl(H_2_O)_6_	30.5 g/L	0.4	1.1
(NH_4_)_2_CO_3_	48 g/L	0.5	—
HCl	6 mol/L	1.3	0.7

In the gastric digestion stage, 1 g of DES and ethanol-extracted MOLLs phenolic extracts were weighed, and 8 mL of gastric digestion reserve solution, 0.42 mL of pepsin (2,000 U/mL), and 27.5 μL of CaCl_2_ solution (44.1 g/L) were added. The pH was adjusted to 3.00, and ultrapure water was added to 10 mL. Then, the samples were mixed well and placed in a 37°C shaker in the dark, after which the reaction was shaken for 2 h. After gastric digestion, the reaction was completed with the following steps. At the end of gastric digestion, the samples were removed.

During the intestine digestion stage, 4 mL of reserve solution was added for intestinal digestion, 2.5 mL of trypsin (100 U/mL), 1.5 mL of bile salt (pig) (200 mg/mL), and 20 μL of CaCl_2_ solution were added, and the pH was adjusted to 7.00. Then, ultrapure water was added to the mixture to a volume of 20 mL. The mixture was fully mixed and incubated at 37°C with constant temperature shaking for 2 h in the dark. After that, the mixture was centrifuged at 4°C (10,000 r/min). After 10 min, the supernatant was removed and stored at −20°C for measurement.

##### 2.8.2.2 Detection of DPPH and FRAP

The DPPH radical scavenging rate was detected by modifying the assay protocol of Romanet et al. (17) as appropriate. To prepare the DPPH working solution (0.25 mmol/L), 50.72 mg of DPPH reagent was weighed, dissolved in 50 mL of ethyl alcohol, and allowed to stand at room temperature in the dark for 12 h. The undigested sample served as the control. For each sample, 1 mL of the sample (at 1 mg/mL) was mixed with 9 mL of DPPH solution (*A_i_*), 1 mL of ethyl alcohol was mixed with 9 mL of DPPH solution (*A*_0_), and 1 mL of ethyl alcohol was mixed with 9 mL of the sample (*A_j_*). All the mixtures were allowed to react for 1 h in the dark at room temperature, and subsequently, their absorbance values were measured at 517 nm.

FRAP was performed according to the method of Khadhri et al. (18). The following solutions were used: 1% (w/v) aqueous K_3_FeC_6_N_6_ solution, 10% (w/v) aqueous trichloroacetic acid solution, 0.1% (w/v) aqueous iron chloride solution, and phosphate buffer. Then, 0.25 mL of the sample to be tested (1 mg/mL), 0.25 mL of phosphate buffer solution and 0.25 mL of K_3_FeC_6_N_6_ aqueous solution were added to a test tube. The test tube was submerged in a water bath heated to 50°C for 20 min and then quickly removed and cooled. Then, 0.25 mL of 10% trichloroacetic acid aqueous solution was added. The above mixture was centrifuged at 3,000 r/min for 10 min. Following centrifugation, 0.5 mL of the supernatant, 0.4 mL of ultrapure water, and 0.1 mL of aqueous iron chloride solution were added. 0.5 mL of the supernatant was removed, 0.4 mL of ultrapure water and 0.1 mL of aqueous iron chloride solution were added, and the solution was mixed thoroughly. The absorbance at 700 nm was measured.

DPPH and FRAP detection are using Tolox as a standard, the calibration curve equations is: *y* = 0.7070*x* − 0.016, *R*^2^ = 0.9976.

### 2.9 Statistical analysis

Origin 2021, GraphPad 9.5, and Design-Expert 13 were used for graphic processing, and SPSS 23.0 was used for statistical analysis. Duncan’s test was applied to compare the data at the 95% confidence level. The experiment was conducted three times, and the results are presented as the mean ± standard deviation.

## 3 Results and discussion

### 3.1 Characterization of the DESs

HBDs are interspersed between HBA molecules and interact to form DESs (19). Studying the physical properties of DESs is crucial for their advancement and application. Density is an essential physical property of liquids. Table 1 presents the densities of DESs, which, except for urea, are lower than those of water. This reveals a relationship between the density of DESs and the number of HBDs present (20). Furthermore, the affinity of DESs for water also affects their density: hydrophilic DESs are unstable in aqueous solution, but hydrophobic DESs are more stable in such a medium. Hydrophobic DESs are more advantageous for extraction applications because they can extract more nonpolar bioactive compounds (21, 22). Research has demonstrated that the density of hydrophobic DESs is lower than that of water, whereas that of hydrophilic DESs is similar to or greater than that of water (23). These results suggest that low-density DES solvents can be used to improve the extraction efficiency of phenolic compounds from MOLLs.

**Figure 1:**
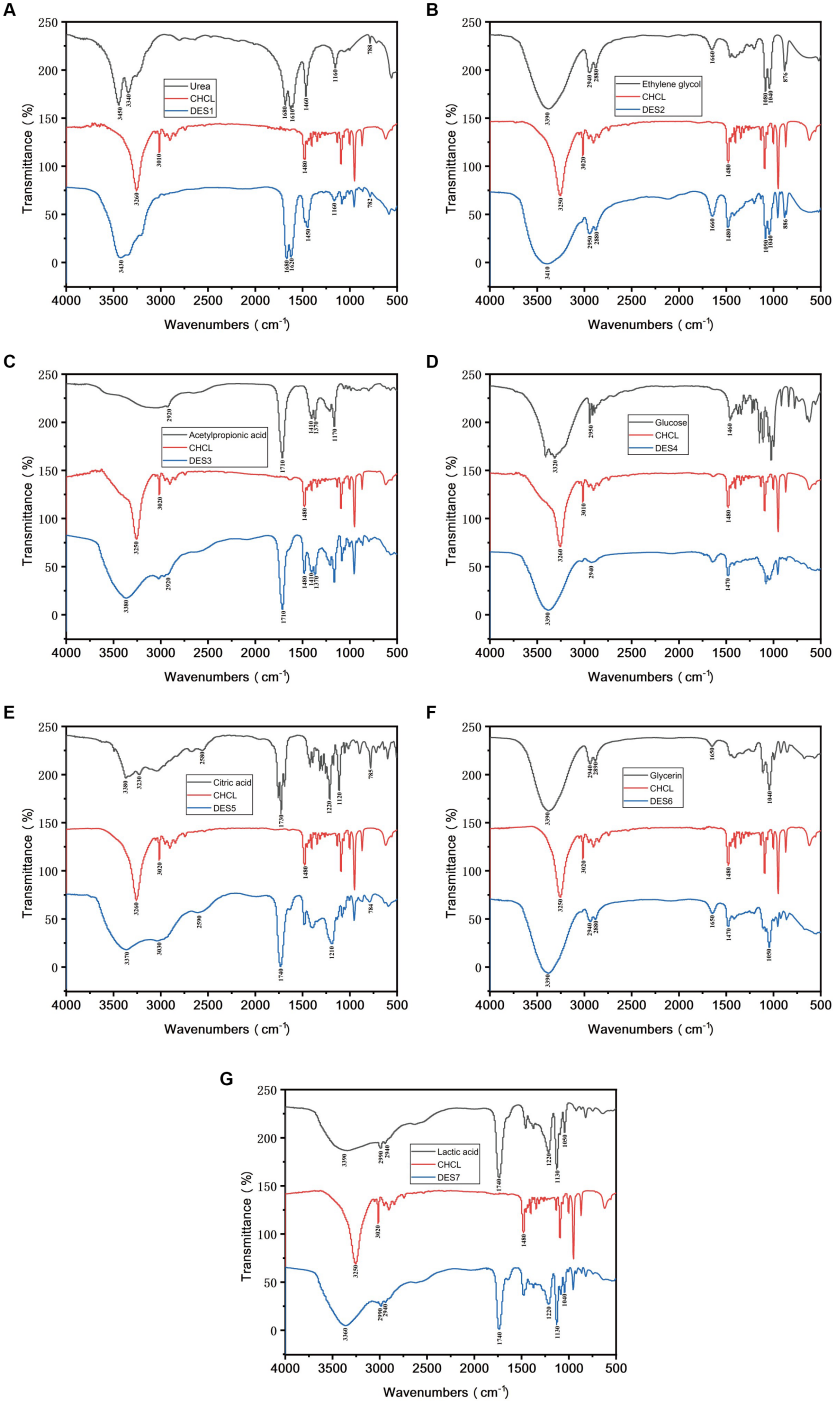
The FT-IR of DESs. **(A-G)**: DES1-DES7.

FT-IR was used to characterize the prepared DES solutions to investigate the solvent synthesis, and the Fourier spectra of DES1-DES7 are shown in Figure 1, respectively. From Figure 1, it is clear that the –OH absorption peak of ChChl is near 3,260 cm^−1^, which suggests that intramolecular hydrogen bonding exists in ChChl. After adding different HBDs, the –OH absorption peaks of the DES solvent shifted to 3,430 cm^−1^, 3,410 cm^−1^, 3,380 cm^−1^, 3,390 cm^−1^, 3,370 cm^−1^, 3,390 cm^−1^, and 3,360 cm^−1^. The shift of the –OH absorption peak in the spectrum of the DESs suggests that the hydrogen bonding ability of ChChl weakens with the addition of HBD. As a result, a part of the electron cloud of the oxygen atom shifts to form a stronger hydrogen bond with the hydrogen bonding donor, which lowers the bonding constant, causing the –OH absorption peak to shift and resulting in the formation of a broader peak near 3,300 cm^−1^ in DESs, which correlates to the creation of additional hydrogen bonds (24), and it also indicates that the DESs were hydrogen bonded to the HBD during the process of ChChl formation (25). The results show that the synthesized DES solvents with different hydrogen bond donors and ChChl retain the characteristic peaks of the hydrogen bond donors, indicating that the functional groups in the solvent are relatively stable (26). The above results proved that the DESs synthesized by different HBDs had different structures, and the formation of hydrogen bonds in the solvents and the changes in bond absorption peaks predicted differences in the extraction efficiency of the DESs for phenolic compounds.

### 3.2 Evaluation of the efficiency of DESs for the extraction of TPC and TFC from MOLLs

**Figure 2:**
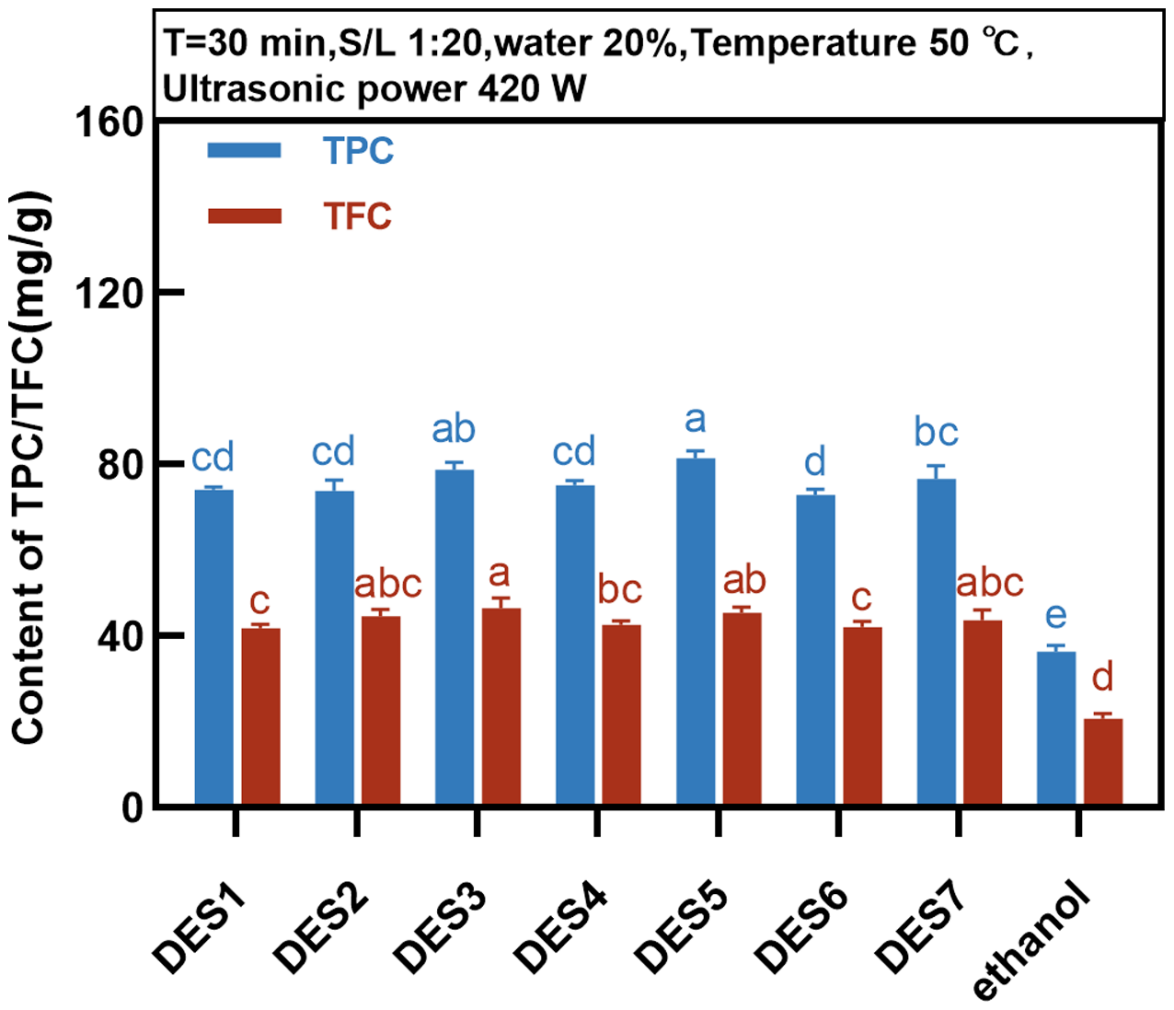
Effect of different deep eutectic solvents on the content of TPC and TFC.

The TPC and TFC in the extracts of MOLLs were detected for the preliminary evaluation of the extraction efficiency of the DESs. DESs with different molar ratios, all with 20% water content, were prepared as shown in Table 1. Phenolic extracts of the MOLLs were prepared using each of the seven DESs and compared with the extracts obtained with a 70% ethanol solution. The extraction fixation conditions were as follows: temperature of 50°C, solid–liquid ratio of 1:20 g/mL, and ultrasonic power of 420 W. The extraction of TPC and TFC from MOLLs by DESs is shown in Figure 2.

The DES solvent can provide protons and accept electrons, which facilitates the formation of intermolecular hydrogen bonding forces, and phenolic compounds can form strong molecular hydrogen bonds and electrostatic interactions between DESs, which enhances phenolic compound solubility (27). As shown in Figure 2, the percentage of phenolic compounds extracted from MOLLs by the seven DESs was greater than that extracted by the solvent 70% ethanol, and all of these percentages were significantly different from that of the 70% ethanol solvent extract.

The extraction rates of TPC and TFC from seven DESs were different due to the solvent polarity caused by the compositions of HBDs and HBA. DESs synthesized from organic acids such as HBDs (DES3, DES5, and DES7) showed higher extraction rates of TPC and TFC, with the highest TPC of 81.44 ± 1.64 mg GAE/g for DES5 and the highest TFC of 46.53 ± 2.39 mg RT/g for DES3. Studies have shown that DESs based on organic acids are more effective than those based on sugars and polyols for the extraction of phenolic compounds (28, 29), which is similar to the results of the present study. Compared to the remaining DESs, DES6 (choline chloride:glycerol) showed a lower extraction rate of phenolic compounds from MOLLs, at 76.63 ± 2.97 mg GAE/g and 43.61 ± 2.40 mg RT/g, which was attributed to the fact that glycerol leads to stronger spatial site resistance, weakening the interaction of chloride ions with target compounds, and thus decreasing the extraction efficiency of phenolic compounds (30). By comprehensively comparing the extraction efficiencies of seven DESs for TPC and TFC in MOLLs, we chose DES-5 (choline chloride: citric acid) as the best extraction solvent for subsequent experimental studies.

### 3.3 Optimization of extraction process parameters by single factor experiment

#### 3.3.1 Solid–liquid ratio

**Figure 3:**
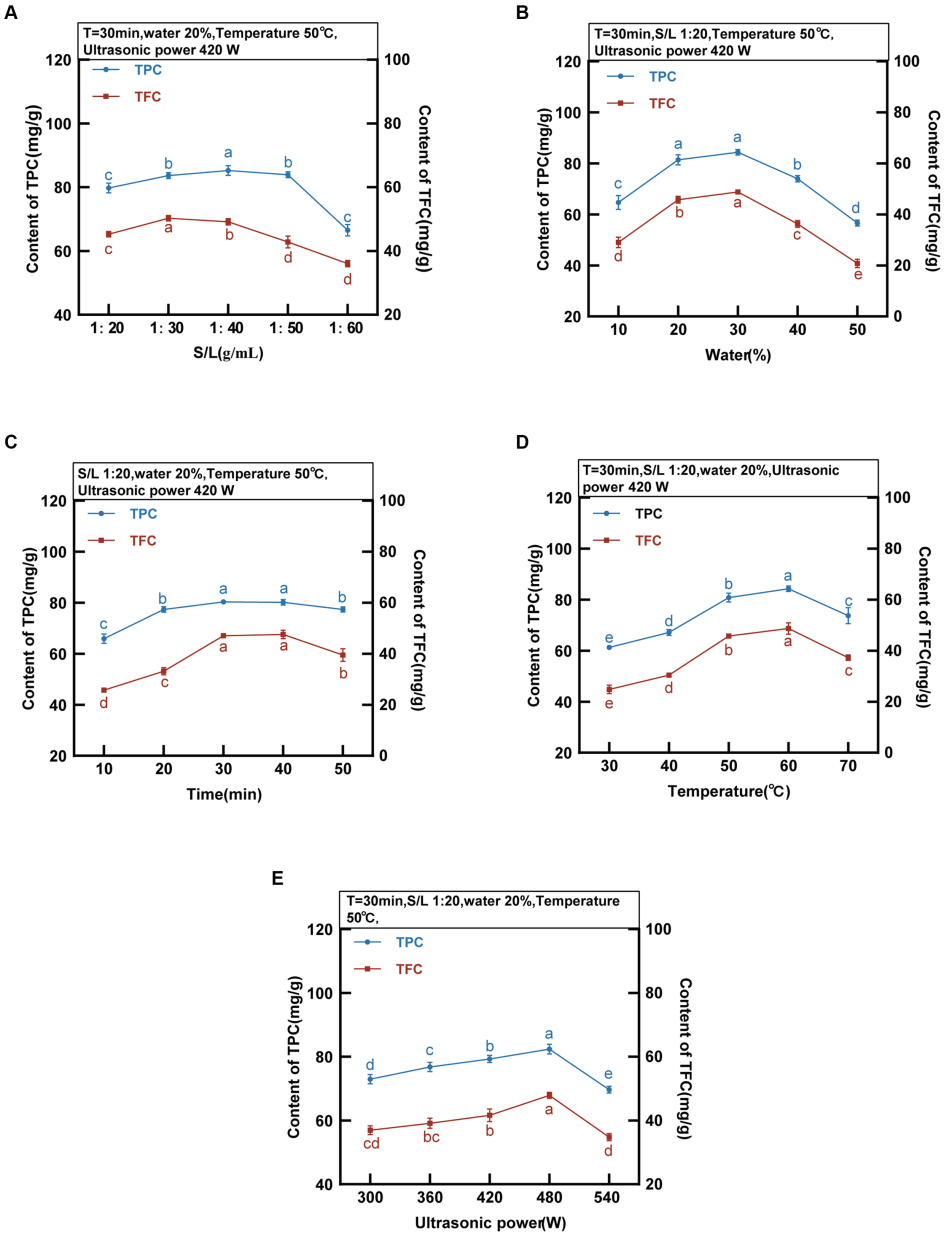
DES-5 Extraction of TPC and TFC from MOLLs at different S/L ratio, water, time, temperature and ultrasonic power.

The ratio of solid to liquid is a critical factor affecting the efficiency of phenolic compound extraction. The TPC and TFC of the MOLLs were detected at various solid–liquid ratios while keeping the following other parameters constant: extraction time of 30 min, water content of 20%, temperature of 50°C, and ultrasonic power of 420 W, as displayed in Figure 3A. The transfer efficiency of mass at the solid–liquid interface changes with the ratio of solid to liquid, and finding the optimal solid–liquid ratio is favorable for extracting phenolic compounds. The extraction of TPC and TFC increased with the solid–liquid ratio between 1:20 g/mL and 40 g/mL and reached its peak at 1:40 g/mL with 85.22 ± 1.53 mg GAE/g of TPC and 50.31 ± 0.87 mg RT/g of TFC. TPC and TFC extraction decreased at liquid ratios between 1:50 g/mL and 1:60 g/mL and reached a minimum at 1:60 g/mL, with 66.50 ± 1.81 mg GAE/g of TPC and 36.01 ± 1.01 mg RT/g of TFC. Since the solid–liquid ratio significantly affects production costs, the solid–liquid ratio was fixed at 1:30 g/mL for all subsequent experiments.

#### 3.3.2 Water content of DES-5

The high viscosity of DESs can be improved by adding an appropriate amount of water, which enhances their surface tension and polarity, resulting in increased mass transfer efficiency and extraction rate (31). The TPC and TFC were detected at various water contents (10–50%) of DES-5 while keeping the other parameters constant: extraction time of 30 min, solid–liquid ratio of 1:20 g/mL, temperature of 50°C, and ultrasonic power of 420 W. The results are shown in Figure 3B. The TPC and TFC increased gradually with increasing water content, reaching maxima of 84.39 ± 1.11 mg GAE/g and 48.85 ± 0.43 mg RT/g, respectively, at a water content of 30%. A suitable water content can regulate the hydrogen bonding force in the DES, which increases the leaching of TPC and TFC (32). The extractions of TPC and TFC gradually decreased at a water content of 30–50%, suggesting that excessive water reduces the interaction of the DES with the phenolic compounds present in MOLLs, leading to a decrease in the leaching of TPC and TFC (33). Rashid et al. (11) investigated the extraction of phenolic compounds from apple pomace using DESs with different water contents and reported that the extraction efficiency of phenolic compounds decreased when the water content of the DES was greater than 30%, which is consistent with the results of the present study.

#### 3.3.3 Extraction time

The ultrasound-assisted extraction process involves considering the extraction time as a crucial parameter. The TPC and TFC were detected at various extraction times (10–50 min) while keeping the other parameters constant: solid–liquid ratio of 1:20 g/mL, water content of 20%, temperature of 50°C, and ultrasonic power of 420 W. The results are detailed in Figure 3C. With increasing extraction time, the solubility of phenolic compounds in DESs within MOLLs increased gradually. The maximum extraction occurred at 30 min, with TPC and TFC values of 80.35 ± 0.90 mg GAE/g and 47.42 ± 0.57 mg RT/g, respectively. The TPC and TFC decreased gradually from 30 to 50 min, reaching 77.36 ± 0.97 mg GAE/g and 39.50 ± 2.48 mg RT/g, respectively. This reduction was due to the degradation of some phenolic compounds caused by high temperatures and prolonged reaction times (34).

#### 3.3.4 Temperature

The extraction efficiency is affected by excessively high temperatures, leading to the decomposition of phenolic compounds. As a result, a temperature range between 30 and 70°C was selected to examine the impact of DES on the extracted TPC and TFC contents. The TPC and TFC were detected at various temperatures while keeping other parameters constant: an extraction time of 30 min, a solid–liquid ratio of 1:20 g/mL, a water content of 20%, and an ultrasonic power of 420 W. Figure 3D displays the results. Within the range of 30–60°C, the TPC and TFC gradually increased before reaching their maximum values at 60°C (84.27 ± 1.08 mg GAE/g and 48.73 ± 2.25 mg RT/g, respectively). The increased temperature resulted in increased molecular kinetic energy within the DES, simultaneously decreasing its viscosity. This strengthening of the solvent-solid interaction caused an increase in phenolic compounds (30). Conditions that utilize ultrasound-assisted extracts have an enhanced cavitation effect due to temperature increases. The combination of thermal and cavitation effects results in an increase in extraction efficiency (35). The extraction content decreased above 60°C. The TPC and TFC at 70°C were 73.75 ± 3.10 mg GAE/g and 37.29 ± 1.09 mg RT/g, respectively. This is attributable to the lower extraction efficiency stemming from the degradation of the TPC and TFC, which are thermally unstable.

#### 3.3.5 Ultrasonic power

The ultrasonic power significantly affects the extraction rate in the ultrasound-assisted extraction method. By adjusting the ultrasonic power to a range of 300–540 W, we observed the effect of DES on the extraction rate of TPC and TFC. The TPC and TFC were detected at different ultrasonic power levels while keeping the following parameters constant: extraction time of 30 min, solid–liquid ratio of 1:20 g/mL, water content of 20%, temperature of 50°C, and the results are shown in Figure 3E. The results indicate that the quantity of TPC and TFC extracted increases proportionally with increasing ultrasonic intensity within the range of 300 W-480 W, achieving the maximum values of TPC and TFC at 480 W, with the highest values being 82.41 ± 1.52 mg GAE/g and 47.89 ± 0.92 mg RT/g, respectively. Increasing the power intensity increased the acoustic wave intensity, which led to a strengthened cavitation effect. The increase in power intensity resulted in the energy collapse of cavitation and the generation of shock waves, thereby increasing the efficiency of phenolic compound extraction (36). When the ultrasonic power was maintained between 480 W and 540 W, increasing the ultrasonic power resulted in a decrease in the TPC and TFC. An increase in power intensity led to an increase in medium temperature, which caused the degradation of phenolic compounds as a result of greater heat dissipation and increased heat sensitivity (35).

### 3.4 Optimization of the extraction process parameters by RSM

#### 3.4.1 Multiple regression results and fitted model analysis

Based on the results of the single factor experiment, the extraction time (*A*), DES water content (*B*), ultrasonic power (*C*), and extraction temperature (*D*) were selected as the optimization factors, and 29 optimization experiments were carried out to optimize the extraction process of phenolic compounds from MOLLs by DES-5 using the BBD method; the four variables and their levels are shown in Table 2. In addition, the data were fitted to a quadratic polynomial regression equation of coded factors with the following polynomial equations of coded factors for TPC and TFC:


$$\begin{array}{c} TPC=86.91-0.5939A-2.15B-2.63C-1.13D+0.4547\mathrm{AB}\\ -\;0.1394\;\mathrm{AC}+0.9929\;\mathrm{AD}-0.8142\;BC+0.4381BD\\ +\;2.11CD-2.55A^{2}-7.24B^{2}-6C^{2}-5.71D^{2} \end{array}$$

$$\begin{array}{c} TFC=50.47–0.5524A-2.23B-2.53C-1.09D+0.857\mathrm{AB}\\ -\;0.4556\;\mathrm{AC}+1.1\;\mathrm{AD}-0.0234\;BC+0.5017BD+1.91CD\\ -\;2.46A^{2}-6.82B^{2}-5.59C^{2}-5.26D^{2} \end{array}$$

To assess the validity of the quadratic polynomial model, we used ANOVA. As shown in Table 4, the TPC and TFC models are significant (*p* < 0.0001), and the lack of fit is not significant (*p*_TPC_ = 0.4442 > 0.05; *p*_TFC_ = 0.5005 > 0.05), indicating that the proposed regression equations have a small error in fitting the experimental data and that the independent variables have a significant effect on the results (37). *R*^2^ (0.9536) and *R*^2^_Adj_ (0.9073) for TPC and *R*^2^ (0.9543) and *R*^2^_Adj_ (0.9085) for TFC, indicating a good model fit, and the reproducibility of the model was demonstrated by the coefficient of variation (CV), which was 2.08 and 3.62 for TPC and TFC, respectively; in general, the acceptable coefficients of variation are usually less than 20 (38). The results of the study support model reproducibility. The degree of influence of first-order linear effects on TPC and TFC decreased in the order *C* > *B* > *D* > *A*. Second-order quadratic effects (*CD*) had a significant effect on TPC and TFC (*p*_TPC_ = 0.0209 < 0.05; *p*_TFC_ = 0.0254 < 0.05).

**Table 4:** Analysis of variance (ANOVA) of responses for TPC and TFC.

Source	TPC					TFC				
	Sum of squares	df	Mean square	*F*-value	*p*-value	Sum of Squares	df	Mean square	*F*-value	*p*-value
Model	758.6	14	54.19	20.57	<0.0001	679.26	14	48.52	20.86	<0.0001
*A* Time (min)	4.23	1	4.23	1.61	0.2256	3.66	1	3.66	1.57	0.2301
*B* Water (%)	55.64	1	55.64	21.13	0.0004	59.74	1	59.74	25.69	0.0002
*C* Ultrasonic power (%)	82.95	1	82.95	31.49	<0.0001	76.85	1	76.85	33.04	<0.0001
*D* Temperature (°C)	15.4	1	15.4	5.85	0.0298	14.34	1	14.34	6.17	0.0263
*AB*	0.827	1	0.827	0.314	0.5841	2.94	1	2.94	1.26	0.28
*AC*	0.0777	1	0.0777	0.0295	0.8661	0.8303	1	0.8303	0.357	0.5597
*AD*	3.94	1	3.94	1.5	0.2413	4.82	1	4.82	2.07	0.172
*BC*	2.65	1	2.65	1.01	0.3327	0.0022	1	0.0022	0.0009	0.976
*BD*	0.7677	1	0.7677	0.2915	0.5978	1.01	1	1.01	0.4329	0.5212
*CD*	17.84	1	17.84	6.77	0.0209	14.55	1	14.55	6.25	0.0254
*A*^2^	42.33	1	42.33	16.07	0.0013	39.22	1	39.22	16.87	0.0011
*B*^2^	340.36	1	340.36	129.22	<0.0001	301.72	1	301.72	129.73	<0.0001
*C*^2^	233.79	1	233.79	88.76	<0.0001	202.79	1	202.79	87.19	<0.0001
*D*^2^	211.36	1	211.36	80.25	<0.0001	179.22	1	179.22	77.06	<0.0001
Residual	36.87	14	2.63			32.56	14	2.33		
Lack of fit	27.99	10	2.8	1.26	0.4442	23.94	10	2.39	1.11	0.5005
Pure error	8.88	4	2.22			8.62	4	2.15		
Cor. total	795.47	28				711.82	28			

#### 3.4.2 Effect of the DES-5 extraction parameters on the percentage of phenolic compounds extracted from MOLLs

**Figure 4:**
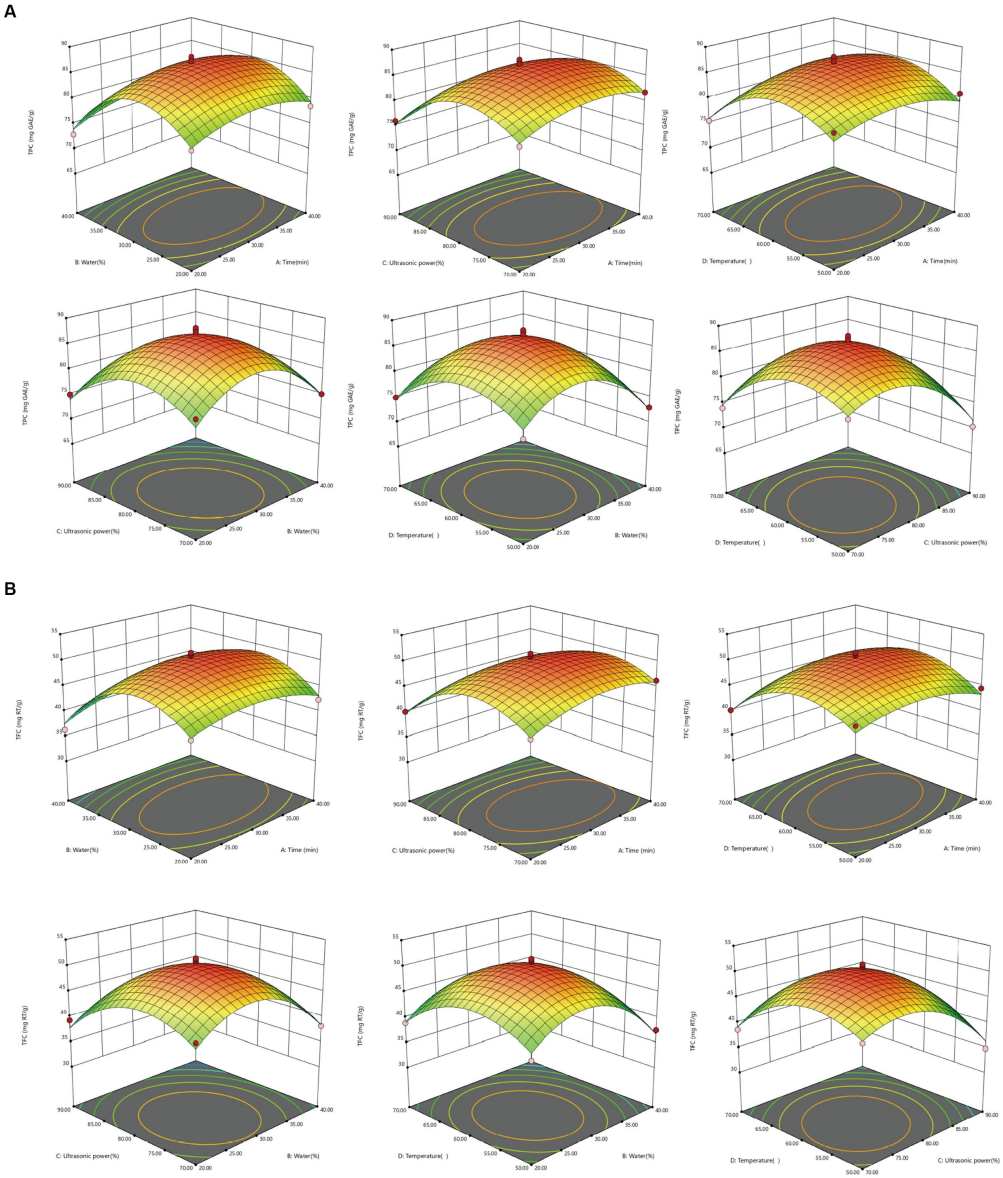
Response surface diagram of the interaction of various factors on extraction content of TPC and TFC from MOLLs. **(A)** TPC, **(B)** TFC.

Parameter optimization of the extraction conditions is necessary to effectively reduce the production cost by obtaining a higher extraction rate under suitable conditions. 3D surface response plots, shown in Figure 4 for TPC and TFC, illustrate the relationship between the extraction parameters and extraction amount. The 3D graph is a spherical structure that can better present the interaction between different factors. In the 3D surface response plots, the greater the slope inclination, the stronger the interaction between the two factors.

As can be seen intuitively in Figure 4, *CD* interaction is the strongest (*p*_TPC_ = 0.8661 > 0.05; *p*_TFC_ = 0.5597 > 0.05) and *AC* (*p*_TPC_ = 0.0209 < 0.05; *p*_TFC_ = 0.0254 < 0.05) interaction is weak. The difference analysis in Table 4 also shows the same result. During the extraction of phenolic compounds from plant material using ultrasound-assisted extraction, the amplitude should be carefully controlled to avoid overexposure of the plant material, which might lead to the degradation of bioactive compounds (39). In turn, the increase in TPC and TFC depends on the appropriate amplitude, which is essential for achieving high levels of bioactive compounds. The results of this study showed that ultrasonic power (*p* < 0.0001) was the most significant factor affecting the extraction of TPC and TFC. The 3D surface response plots of the interactions of *AC*, *BC*, and *CD* revealed a trend where the TPC and TFC first increased and then decreased slightly with increasing ultrasonic power at a fixed immersion time, water content, and temperature. Furthermore, the water content of the DES-5 solvent was also a significant factor (*p*_TPC_ = 0.0004 < 0.01; *p*_TFC_ = 0.0002 < 0.01) affecting the extractions. The study also revealed that under certain conditions of ultrasonic power, temperature, and extraction time, increasing the solvent water content increased the extraction of TPC and TFC. However, it was also observed that using too much water in the solvent was still unfavorable for the extraction of phenolic compounds. An optimum temperature can enhance the mass transfer efficiency of bioactive compounds, ultimately improving the extraction outcome (40). The interaction effects between *AD*, *CD*, and *BD* indicated that the TPC and TFC increased and then decreased with increasing temperature when the extraction time, water content, and ultrasonic power were consistent. The highest value was achieved at a temperature of 60°C, which corresponded with the results of the single-factor experiment.

Following multiple numerical optimizations, the following optimal process conditions were determined: 30.787 min of extraction time, 28.492% water content, 465.264 W of ultrasonic power, and a temperature of 58.542°C. To simplify the experimental procedures, the following conditions were used: 30 min of extraction, 28% water content, 480 W of ultrasonic power, and 58°C. The results are shown in Table 5, the validation indicated that small variations and differences between the experimental responses and predicted values under the optimal conditions confirmed the validity of the BBD design model’s optimal conditions.

**Table 5:** Validation results of RSM.

	Predicted results	Actual results
TPC (mg GAE/g)	87.467	86.92 ± 1.34
TFC (mg RT/g)	51.08	49.73 ± 0.85

### 3.5 High-temperature and UV stabilization studies of phenolic extracts

Phenolic compounds have one or more hydroxyl groups in their structure. They become less stable as the number of hydroxyl groups increases (41). Phenolic compounds are mainly affected by temperature and light during food processing and storage. The chemical structure of phenolic compounds changes due to high temperature and continuous light, leading to their instability (42).

**Figure 5:**
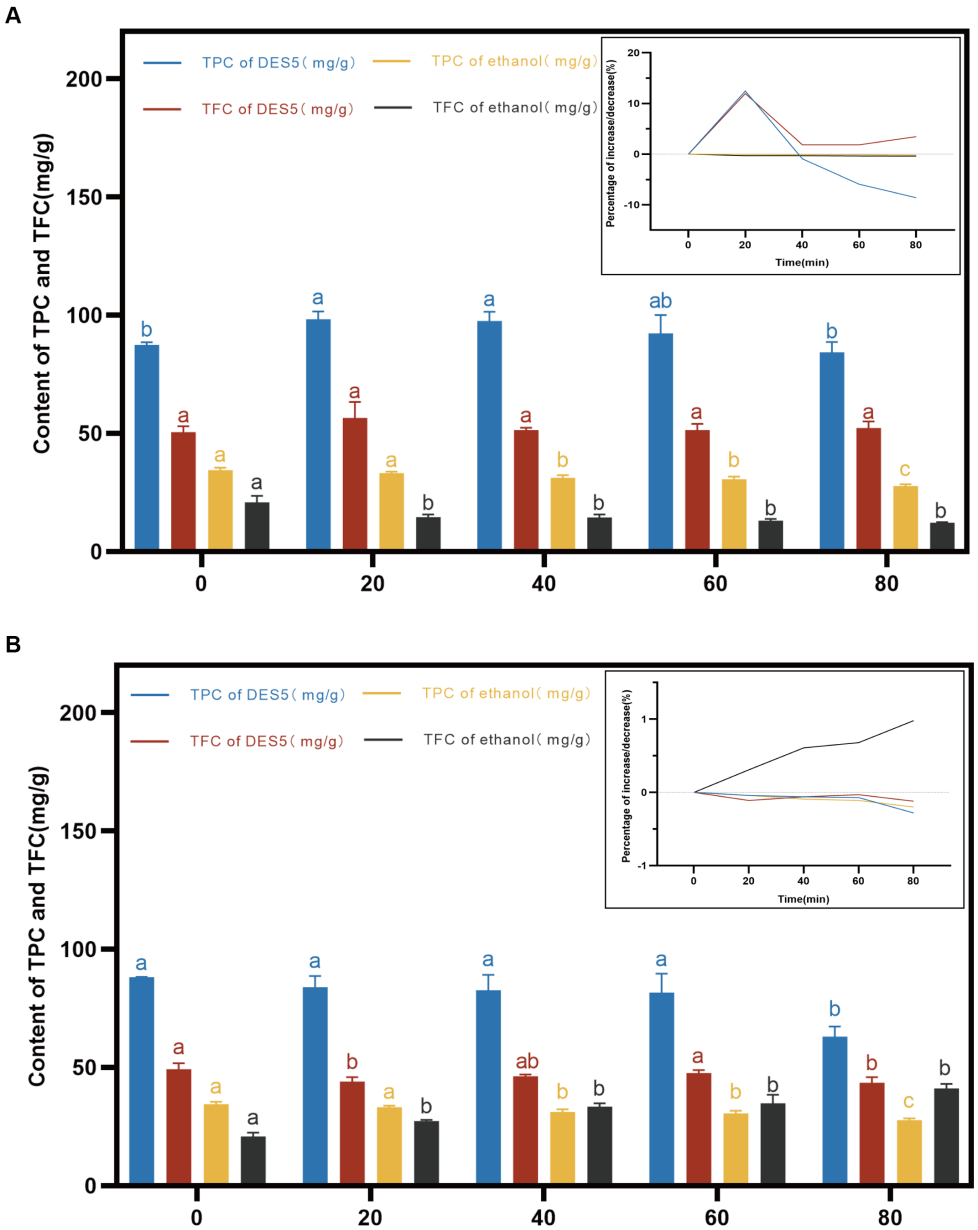
Effects of high temperature and UV intervention at different time on TPC and TFC in DES-5 and ethanol. **(A)** high temperature, **(B)** UV.

Figure 5A illustrates the trend and phenolic content of the DES and ethanol extracts maintained at 100°C for 0–80 min. As depicted in Figure 5A, the phenolic content of both the ethanol and DES extracts decreased to below the initial value after 80 min at 100°C. In contrast to the DES extract, the ethanol extract displayed a milder trend. The TPC and TFC of the DES extract increased from 0 to 20 min and then decreased gradually. A notable difference was observed between the TPC at 80 min and 0 min, while the TFC remained stable after 40 min. Research has demonstrated that high-temperature conditions can cause certain phenolic compounds to undergo distinctive isomerization, increasing the phenolic content. However, over time at high temperature, some phenolic compounds are lost due to thermo-oxidative degradation, leading to a decrease in phenolic content (41, 43).

Phenolic compounds are sensitive not only to temperature but also to degradation by light. Figure 5B illustrates that, over a time span of 0–80 min, the DES and ethanol extracts were subjected to UV radiation, resulting in a gradual decrease in the TPC and TFC in the DES extracts and in the TPC in the ethanol extracts due to the high photosensitivity and unsaturated bonds of phenolic compounds, which are gradually broken down by direct UV radiation, leading to the partial degradation or polymerization of the phenolic active compounds (42). Notably, the TFC in the ethanol phenolic extracts increased gradually with increasing UV radiation time and exhibited significant differences between 80 min and 0 min of radiation. This increase may be due to structural changes in the extract’s compounds, although no relevant studies have been conducted yet.

Overall, high temperature had a greater effect on the stability of the TPC in the DES extracts, but high-temperature heating for a shorter period of time (20 min) increased the TPC and TFC content in the DES extracts. High temperature also decreased the content in the ethanol extracts compared to that in the ethanol extracts, but the trend was flat. UV radiation had less of an effect on phenolic extracts than did high-temperature treatment, but UV radiation increased the TFC content in the ethanol extracts. Therefore, proper heating and UV radiation can increase or decrease the phenolic content to a certain extent, which indicates that phenolic compounds are unstable, and their instability also predicts better bioactivities, such as free radical scavenging ability. However, methods such as low temperatures, light avoidance, phenolic modification, or the use of novel carrier systems need to be employed to prevent phenolic compound degradation during processing and storage.

### 3.6 Effect of *in vitro* simulated digestion on the phenolic content and antioxidant activity of MOLLs

Phenolic compounds have a variety of physiological activities that are beneficial to human health. However, phenolic compounds need to be absorbed through the digestive system and then act on target organs and cells to exert their physiological effects. INFOGEST 2.0 *in vitro* static simulation of gastrointestinal digestion was used to study the changes in the content of DES and ethanol extracts at different stages of digestion and to investigate the antioxidant activity of phenolic compounds after simulated digestion.

**Figure 6:**
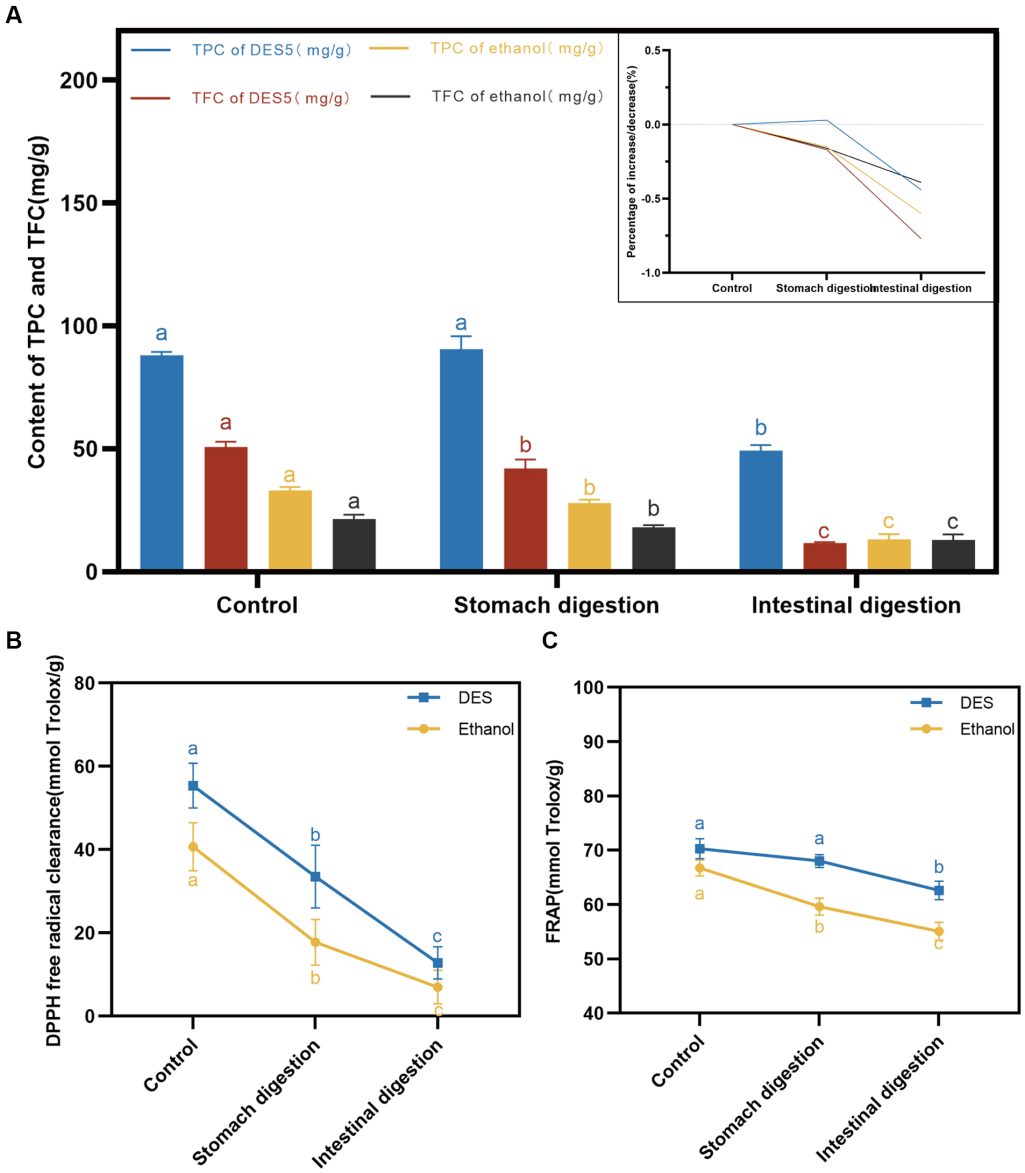
DES-5 extraction methods on in vitro simulated digestion of TPC and TFC. **(A)** content of TPC and TFC, **(B)** DPPH radical scavenging rate, **(C)** FRAP.

The TPC and TFC of the DES and ethanol phenolic extracts decreased following digestion with the gastric and intestinal fluids, as illustrated in Figure 6A. In the DES extracts, the TPC did not decrease significantly after hydrolysis by pepsin (*p* = 0.4025 > 0.05). This may be attributed to the weak acidity of the simulated gastric fluid, which provides a better protective effect on phenolic acids in the extracts (44). However, in the ethanol extract, the TPC decreased significantly in the simulated gastric fluid (*p* = 0.0378 < 0.05), and research has demonstrated that different categories of phenolic compounds undergo alterations after simulated digestion in the gastrointestinal tract (45). Furthermore, the contents of phenolic extracts of DES and ethanol decreased after intestinal simulated fluid digestion, exhibiting significant differences (*p* < 0.0001). The decrease in TFC was more pronounced for the DES extracts. Tenore et al. (46) examined the changes in tea phenolic compounds *in vitro* during digestion and discovered that phenolic compounds were susceptible to the alkaline environment of the intestinal fluid. Flavonoids, in particular, are vulnerable to oxidation and degradation under mildly alkaline conditions in the intestinal fluid.

Antioxidant capacity is closely related to the content of phenolic compounds, with flavonoids possessing superior antioxidant properties (47). The antioxidant activity of the phenolic extracts was determined using DPPH and FRAP methods after treatment with simulated gastrointestinal digestive fluids. The results are shown in Figures 6B,C. During the simulated digestion process, the DPPH scavenging capacity and FRAP of the DES and ethanol phenolic extracts decreased gradually. After digestion by intestinal fluids, the antioxidant activity of the DES phenolic extracts was superior to that of the ethanol extracts, which confirms our previous studies on the relative instability but better DPPH scavenging capacity and ferric ion reducing antioxidant power of DES phenolic compounds when compared to ethanol extracts.

## 4 Conclusion

UAE-DES is a simple and effective method for extracting phenolic compounds from MOLLs. The extraction efficiency of the seven prepared DESs was better than that achieved using 70% ethanol. Optimizing the operating parameters using single-factor experiments and response surface methods improved the extraction efficiency of DES-5 for TPC and TFC. Intervening phenolic extracts using high temperature and UV radiation showed that the extracts obtained by DES-5 were less stable. After *in vitro* simulated digestion, the DES-5 extracts exhibited good DPPH free radical scavenging and ferric ion reducing antioxidant power. This study provides a foundation for developing an efficient method for extracting phenolic compounds through DESs. The results showed that DESs enhanced the extraction efficiency of phenolic compounds from MOLLs, demonstrating their potential as substitutes for organic solvents in the extraction of bioactive substances.

## Data availability statement

The original contributions presented in the study are included in the article/supplementary material, further inquiries can be directed to the corresponding authors.

## Author contributions

ZW: Formal analysis, Writing – original draft. YT: Conceptualization, Funding acquisition, Project administration, Writing – review & editing. MY: Writing – review & editing. JY: Formal analysis, Writing – review & editing. YW: Data curation, Writing – review & editing. LT: Conceptualization, Writing – review & editing. JS: Conceptualization, Writing – review & editing. CS: Investigation, Supervision, Writing – review & editing.

## Footnotes

^1^ http://www.nhc.gov.cn/
